# The fossilization of randomized clinical trials

**DOI:** 10.1172/JCI158499

**Published:** 2022-02-15

**Authors:** Nigel S. Paneth, Michael J. Joyner, Arturo Casadevall

**Affiliations:** 1 Department of Epidemiology and Biostatistics, and Pediatrics and Human Development, Michigan State University, College of Human Medicine, East Lansing, Michigan, USA.; 2 Department of Anesthesiology and Perioperative Medicine, Mayo Clinic, Rochester, Minnesota, USA.; 3 Department of Molecular Microbiology and Immunology, Johns Hopkins School of Public Health, Baltimore, Maryland, USA.

**Keywords:** Therapeutics, Statistics

The recognition, in the middle of the 20th century, that the randomized, controlled trial (RCT) was the preferred approach to determining the efficacy of medical interventions was revolutionary. Masking, placebos, and randomization were acknowledgments that investigator bias and advocacy had long been interfering with the assessment of efficacy in medicine. The RCT thus served as a welcome antidote to vacuous claims of treatment success. As early as the 1950s, RCT evidence showed that several commonly recommended treatments were not only ineffective, but harmful (1).

In the ensuing decades, the RCT steadily ascended to the top of the evidence hierarchy in medicine, eventually becoming so dominant that evidence not derived from an RCT came to be viewed in some circles as unusable for clinical or policy decisions. This perspective was further codified by the near-absolute requirement of having one or more RCTs demonstrate the superiority of any new drug or biologic for it to be granted FDA approval. While the 1962 law encoding these requirements did not actually specify the RCT as the only possible source of effectiveness data (2), “ultimately, it was the randomized, double-blinded, placebo-controlled experiment which became the standard by which most other experimental methods were judged” (3). This requirement promoted the development of large, well-funded units devoted to the design and execution of RCTs in nearly every major drug company.

In parallel, a thriving academic industry emerged to address every imaginable component of the RCT process, from randomization schemes to stopping rules to strictures on analysis — fueling, and being fueled by, the evidence-based medicine movement. Educational programs in clinical research focused heavily on the statistical features of RCT design and analysis, further entrenching the RCT as *the* epistemic instrument of medicine. The RCT movement became so intensely focused on method that some key drivers of biomedical science — curiosity, hypothesis formulation, biological rationale, clinical value — to some extent fell by the wayside.

The RCT has now become an unassailable, formula-based, intensely regulated, often inflexible methodology that is often divorced from its clinical and biological roots. Useful guidelines have turned into absolute fiats that can defeat the purpose for which they were intended. How did a critically important development in medicine become an impediment to progress? Although many concerns could be raised, we focus on six core problems of RCTs as they are now conducted: (a) the divorce from biology and clinical experience; (b) the difficulty in avoiding type 2 errors; (c) the insistence on singular trial outcomes; (d) the regulatory burden; (e) the advantage of the pharmaceutical industry; and (f) the eclipse of other forms of evidence.

## Divorcing trial methodology from biology and clinical experience

Trial guidelines are replete with rules guiding every conceivable question as to design and analysis but leave the most fundamental question of any RCT — the biological and clinical hypothesis — up for grabs. As a result, we can have statistically impeccable trials that examine the wrong intervention using the wrong dosage in the wrong population.

Antibody therapies in the form of immune serum and convalescent plasma have been used to treat acute infectious diseases for 130 years, and a voluminous early literature testifies to the fact that this therapeutic modality is effective only when sufficient specific antibody is used and only when treatment is provided early in disease (4). Yet, most RCTs of COVID-19 convalescent plasma (CCP) have studied hospitalized patients days or weeks after disease onset, who were often severely hypoxemic and at times even on ventilators. Such an approach flies in the face of all that is known of the biology and clinical effects of antibody therapies. When RCTs produced no evidence of reduced mortality under the conditions of these experiments, the unfortunate conclusion drawn by many was that CCP does not work at all.

## The type 2 error problem

The great strength of the RCT is that it minimizes type 1 errors — declaring an ineffective treatment effective. But as a tool to avoid type 2 errors — declaring an effective treatment ineffective — the RCT is problematic. The error threshold in RCTs is conventionally four times more stringent for the type 1 error than the type 2 error (typically 0.05 compared with 0.2). Chalmers et al. described the impact of the type 2 error in their classic article on anticoagulants in myocardial infarction (5). Although most trials found reductions in mortality of 25% to 30%, a lack of significance led the trialists to declare the findings null, even though such a mortality reduction would clearly be of value. The reanalysis of these data by Chalmers is credited with revitalizing anticoagulant use in myocardial infarction, leading to the conduct of larger trials that showed the efficacy of anticoagulants that are now universally accepted as a component in the treatment of this disorder.

Although a negative trial only indicates that the treatment does not work in the specific circumstances under which it was tested, when a trial is terminated early because of a statistical judgment that further enrollment is unlikely to show a statistically significant finding, the term “futility” is used. Intended to refer only to the continuation of the particular trial in question, the term powerfully suggests that the intervention is entirely useless.

## Insistence on examining a single trial outcome

An example of the methodologic rigidity of current trial analysis is the insistence on trials having only one primary outcome. This restriction is at first glance sensible, because it is easy to cherry pick the results that seem favorable to the hypothesis under study. But this constraint has spawned a profusion of ways to combine outcomes that are disparate, of uneven weight, and not sensible to combine on a biological basis. Some trials in cardiac disease carefully construct the outcome to be singular by combining deaths and lesser, nonfatal cardiac outcomes. If the study finds fewer nonfatal cardiac outcomes but no change in deaths, the package insert on the medication can misleadingly say “has been shown to decrease the rate of a combined endpoint of death, new myocardial infarction, or refractory ischemia/repeat cardiac procedure,” implying a favorable effect on mortality (6). At the same time, we have trials in newborns, in which the insistence on combining death and disability into a single outcome allows an important beneficial effect on disability to be hidden in the study metric that resulted from an insistence on combining death and disability to ensure that there is just one outcome of the trial (7).

A parallel stricture is the absolute prohibition of subset analysis, a stricture that is unwarranted when biological plausibility indicates that the subset comparison is relevant. In most trials of CCP, participants treated early in the course of illness did better with CCP than did people treated later, as would be expected with passive antibody treatment. But the strict prohibition of subset analysis, as in, for example, the trial by Menichetti et al., led to minimizing the informative finding that CP recipients with a more favorable oxygen profile (PaO_2_/FiO_2_ ratio >300 mmHg) experienced respiratory worsening or death less than half as often as the controls (*P =* 0.11). Shown in a line in a table, the finding was mentioned neither in the abstract nor the discussion (8), which also failed to mention that the trial just missed the primary endpoint for plasma efficacy, with a *P* value of 0.06.

## The regulatory burden

Consent forms that participants must sign are now several pages long and do little to fully convey the meaning of the trial, but rather seem largely intended to protect the institution conducting the trial from legal reprisals, whatever they are imagined to be. Although the NIH is currently trying to unify ethics review standards and authority, local institutional review boards (hospitals, universities) take their turn reviewing procedures in multicenter trials, each suggesting additional constraints. While this dilemma is true for all clinical research, clinical trials generally receive greater scrutiny than do observational research studies. These review processes inevitably take a toll on the pool of individuals willing to participate in trials, with each step yielding an increasingly refined participant population — more educated, higher income, less constrained by the needs of daily life. The generalizability of the trial findings suffers greatly from these restrictions.

## The special advantages of drug company trials

The resources required to set up a properly powered RCT in the academic world can be daunting. While the NIH has modest funds available for early phases of trial work, obtaining funding for the kind of phase III trial that convincingly provides evidence of effectiveness requires the development of a complex grant application that incorporates both preliminary data and an execution plan including details of the study sites and their capacities. This development process takes months to years and then is followed, even under the best of circumstances, by a lag of nearly a year between grant application submission and funding.

By contrast, the pharmaceutical industry’s well-supported clinical trial units have resources, including trained personnel, that can be drawn upon as soon as a need arises, a capacity that ensured that both Pfizer and Moderna could complete and publish their COVID-19 vaccine trials less than a year from the onset of the pandemic in the United States.

The built-in, trial-focused assets of industry place RCTs of therapeutic agents that do not fit into the drug paradigm at a major disadvantage. Consider the trials of two passive antibody therapies during the current pandemic — monoclonal antibodies (mAbs) and CCP. The first is a manufactured product to be sold at a profit; the second is a product derived from voluntary donations and distributed by nonprofit blood banks.

The industry-supported RCTs of mAbs and CCP differed greatly. The mAb trials were conducted rapidly and nearly entirely in outpatients, with resources from Eli Lilly (9–11) and GlaxoSmithKline (12). Two trials principally supported by Regeneron acknowledged some federal support (13, 14). These trials all showed some benefit from mAb treatment. The results of two mAb trials were published, like those of the Pfizer vaccine trial, before the end of 2020, the first year of the pandemic.

By contrast, nearly all CCP plasma trials were mounted in hospitalized patients, reflecting the clinical urgency felt by the medical community to help severely ill patients (15). Although some federal agencies, both in the US and Canada, managed to create sources for more rapid funding of trials under pandemic conditions, all US trials of CCP plasma had to rely on local funds and foundation support, either entirely or in part. None had pharmaceutical industry support. The bulk of these inpatient trials failed to show overall benefit, and, except for small trials stopped early, their results were not published until the fall of 2021. The *New England Journal of Medicine* has published five outpatient mAb trials, but just one outpatient CPP trial, which, like the mAb outpatient trials, showed solid evidence of success (16). But this trial has had no effect on public policy and received scarcely any media coverage.

## Failure to consider sources of evidence other than trials

The US Preventative Services Task Force relies nearly exclusively on RCTs for its recommendations. When good trials are unavailable, as in PSA screening for prostate cancer, no other source of information is consulted. Thus, the decision not to formally recommend PSA screening made no mention of the observation, based on vital data, that US prostate cancer mortality rates had declined by 50%, without any change in incidence, coincident with the introduction of PSA screening (17). Yet vital data have the singular advantage over RCTs of being based on the entire population.

## Conclusion

We see a critical need to rethink how RCTs are designed, conducted, and reported. The heavy emphasis on methodologic exactness needs to be paralleled by an equal emphasis on ensuring that the intervention, its timing, and the population under study are appropriate to the question being asked, taking into account what is known of the biology and clinical features of the condition. We must recognize that current statistical rules make type 2 errors inevitable, leading to mistaken conclusions of lack of efficacy. We must reconsider the rigid insistence on prohibiting hypothesis-driven analysis of trial subsets and the resistance to considering compelling data not emerging from RCTs. Above all, we must stop thinking that the method is the only thing that matters and that subject matter content is irrelevant or infinitely adaptable.

## Version 1 02/15/2022

Electronic publication
